# Experimental and simulation study of inert gas mixture inhibiting coal spontaneous combustion

**DOI:** 10.1038/s41598-024-53979-0

**Published:** 2024-02-21

**Authors:** Xinning Wang, Lei Wang, Weidong Li, Dongyang Liu

**Affiliations:** 1 State Power Construction Investment Inner Mongolia Energy Co., Ltd., Erdos, 017209 China; 2 China Coal Research Institute, Beijing, 100013 China

**Keywords:** Spontaneous combustion of coal, Free radical reaction, Inert mixed gas, High-temperature environment, Inhibit spontaneous combustion, Environmental sciences, Chemistry, Engineering

## Abstract

To explore the mechanism of inhibiting spontaneous combustion of coal by mixed gases, the low-temperature oxidation characteristics of coal under different components of mixed gases were analyzed. ESR and FTIR experiments were used to investigate the effects of different gas mixtures on the activity of coal during low-temperature oxidation and the oxidation reaction of coal surface functional groups. The mechanism of chemical oxygen inhibition of mixed gas was studied by density functional theory. The results show that the larger the CO_2_ component in the mixed gas, the higher the ability to inhibit coal oxidation. The concentration of free radicals in coal under dry air condition is higher than that under inert mixed gas condition during oxidation heating at 30–230 °C. The oxidation ability of –CH_3_, –OH and oxygen-containing functional groups in the mixed gas reaction is inhibited. Through quantum chemistry calculation, it is found that the mixed gas increases the activation energy of free radicals and reduces the heat release of the reaction. This study provides theoretical reference for coal mine thermal disaster.

## Introduction

Spontaneous combustion of coal is one of the main natural disasters in coal mine production, which seriously restricts the sustainable development of coal mine. Coal fire caused by spontaneous combustion of coal has brought huge economic losses to coal mining enterprises, and even endangered the life safety of underground workers^1–3^. To effectively inhibit coal spontaneous combustion, researchers have done a lot of research, and put forward a variety of fire prevention and extinguishing technologies and materials such as yellow mud grouting, water injection, inert gas, inhibitor and gel^4–6^.

Inert gases (N_2_ and CO_2_) are widely used in the prevention and control of coal spontaneous combustion fire in mines because of their advantages such as inerting, cooling, explosion suppression, wide diffusion range, and no damage to instruments and equipment^7–9^. At the same time, many scholars have studied the application of inert gas in mine fire prevention and control. Li^10^ carried out temperature-programmed experiments under different CO_2_ concentrations, and found that CO_2_ with a concentration of more than 30% had obvious inhibition on inerting of coal-oxygen recombination. Liu^11^ carried out temperature-programmed tests on coal samples under different CO_2_ volume fractions, and obtained the best effect of inhibiting coal spontaneous combustion oxidation temperature rise with more than 50% CO_2_. Ma^12^ carried out an experimental study on CO_2_ inhibiting coal oxidation temperature rise. The results show that the greater the concentration of CO_2_, the better the inhibition effect on coal heat release intensity. Guo^13^ studied the experiment of coal spontaneous combustion oxidation temperature rise when inert gases (N_2_, CO_2_) with different concentrations were introduced, and obtained that N_2_ with a concentration of more than 30% had higher inhibition efficiency; The inhibition efficiency of CO_2_ above 20% is higher. To sum up, experts and scholars at home and abroad have obtained the universal law of preventing and controlling coal spontaneous combustion fire, and put forward the technical measures of preventing and extinguishing coal spontaneous combustion fire by using inert gas, and achieved good results. At present, relevant scholars mainly use CO_2_, N_2_, or air mixed with CO_2_ or N_2_ to prevent coal spontaneous combustion, but there is a lack of research on the influence of different components of gas mixture on the low-temperature oxidation process of coal. Cahyadi et al.^14^ found that in O_2_/CO_2_ environment, oxygen-poor combustion of coal has a “ignition delay” phenomenon. Bu et al.^15^ used a fluidized bed to study the ignition behavior of individual coal particles in N_2_/O_2_ and CO_2_/O_2_ atmospheres, and found that when the O_2_ volume fraction was 10%, the ignition delay time in CO_2_/O_2_ atmosphere was much larger than that in N_2_/O_2_ atmosphere. Deng et al.^16^ studied the oxidation characteristics and apparent activation energy changes of carboniferous Permian coal samples in O_2_/N_2_ and O_2_/CO_2_ oxygen-poor atmospheres by using spontaneous combustion oxidation of coal and Fourier transform infrared spectroscopy. The results showed that O_2_ volume fraction decreased or O_2_/CO_2_ atmosphere changed to O_2_/N_2_ atmosphere under the same O_2_ volume fraction. The rate of CO production and oxygen consumption will be reduced.

Coal is composed of inorganic matter and organic matter, and its inner surface structure is complex^17^. In recent years, many scholars have studied the mechanism of inert gas inhibiting coal spontaneous combustion by molecular simulation, which provides theoretical support for goaf fire prevention technology^18–20^. Lou^21^ applied the Giant Canonical Monte Carlo simulation (GCMC) method to analyze the adsorption of O_2_ gas molecules on coal surface from a microscopic point of view, and obtained that the mixed adsorption of CO_2_ and N_2_ gas molecules by coal macromolecules inhibited the adsorption of O_2_ molecules; Cheng et al.^22^ studied the chemisorption of O_2_ on coal surface systematically by using density functional theory (DFT) method. Wu^23^ adopted the GCMC method and injecting power plant flue gas into goaf can store a large amount of CO_2_ and inhibit the first step of coal oxidation spontaneous combustion-physical adsorption of O_2_. In addition to the displacement of gas, the dilution and diffusion of air flow, and the carrying effect of air flow, inert gas also has the effect of inhibiting various elementary reactions in the low temperature oxidation of coal. However, there are few researches on the mechanism of inert gas blocking oxygen from the chemical point of view.

The use of mixed gas can reduce the cost and have better fire prevention effect on inclined mining coal seam. Therefore, it is necessary to further study the inhibition mechanism of mixed gas. In view of this, this paper takes the bituminous coal of Dongrong No.2 Coal Seam as the research object, and uses temperature programmed, ESR and FTIR technology to carry out experiments, and explores the influence of different components of CO_2_ and N_2_ mixed gas on the low-temperature oxidation characteristics of coal; The mechanism of chemical oxygen inhibition of mixed gas is studied by using quantum chemistry theory. It provides theoretical support for coal mine thermal disaster.

## Methods

### Experimental methods

#### Sample information

In this research, the coal samples of Dongrong No.2 Mine in Shuangyashan City, Heilongjiang Province were taken as the research object, and the collected experimental coal samples were sealed with plastic wrap and transported to the laboratory. The particle size of 200 meshes was screened by a grinding machine and a vibrating screen, and then placed in a vacuum drying oven and dried at room temperature for 24 h. The industrial analysis results of coal samples are shown in Table 1.

**Table 1:** Results of industrial analysis of experimental coal samples.

Sample total	Moisture (%)	Ash (%)	Volatile (%)	Sulfur (%)
DR	3.15	19.59	25.68	0.68

#### Low-temperature oxidation experiment

**Figure 1:**
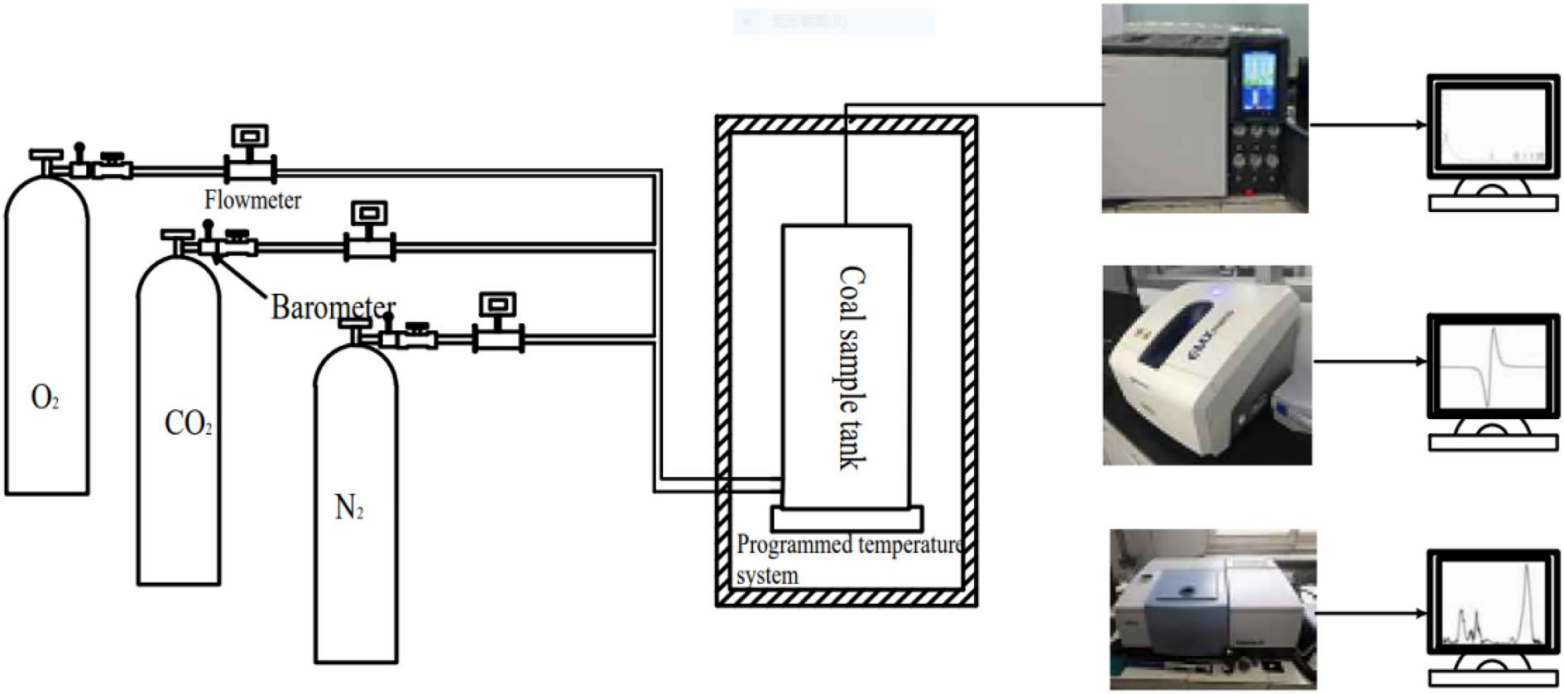
Experimental flow chart.

In the experiment, 40 g of raw coal samples and treated coal samples were put into a coal sample tank respectively, and dry air with a flow rate of 100 mL/min was introduced. The temperature range was 30–230 °C, the heating rate was 1 °C/min, and the CO volume fraction at the primary outlet was recorded when the temperature increased by 10 °C. According to the same analysis method, the inhibition rates of different inhibitors were analyzed in turn. The experimental flow chart is shown in Fig. 1.

#### ESR

Brooke EMXnano Electron Spin Resonance (ESR) was used to determine the change of active groups during low temperature oxidation of coal under different oxidation conditions. Coal samples oxidized to 30, 80, 130, 180 and 230 °C at low temperature were weighed and put into a sample tube to measure ESR spectra. The concentration of free radicals in coal samples was calibrated by Tempol standard sample with known concentration of free radicals. Under the same experimental conditions, the concentration of free radicals in coal samples was indirectly measured by spectrogram area. ESR experimental parameters are shown in Table 2.

**Table 2:** ESR Experimental Parameters.

Central field	Scanning range	Microwave frequency	Power	Modulation frequency
3420G	1000G	9.60 GHz	3.162 mw	100 kHz
Range	Time constant	Conversion time	Receive gain	Scan time
1G	1.28 ms	25 ms	20 dB	50 s

#### FTIR

To investigate the content of active groups in coal oxidation at low-temperature before and after inert gas mixture injection and judge the difference of coal oxidation activity, the TENSOR 27 Fourier Transform Infrared Spectrometer (FTIR) was used for infrared experiment in this study. The coal sample is ground in a mortar to below 200 mesh, and after vacuum drying, the coal and KBr are mixed according to the ratio of 1: 180, and then fully ground and pressed into pieces.

### Simulation method

Gaussian16 software was used for calculation. Hybrid functional B3LYP and 6-311G(6) basis set in density functional theory were used. TS method was used to find the transition state, and it was determined that the structure has only one imaginary frequency, and the vibration mode under this imaginary frequency conforms to the preset bond-breaking trend. The transition state structure is verified by IRC, and the IRC curve is determined to be smooth and complete. The end-point structure at both ends is optimized geometrically to determine that the transition state is connected with reactants and products. The geometrical structure and thermodynamic data of reactants, transition states and products were analyzed to judge the reaction energy barrier.

## Results and discussions

### CO concentration

**Figure 2:**
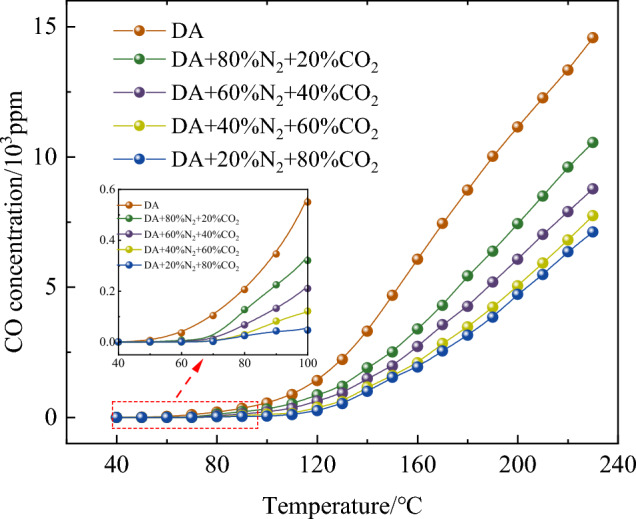
Change of CO concentration under different oxidation conditions.

As can be seen from Fig. 2, compared with dry air, in the process of coal oxidation, the CO emission of coal samples treated by different components of mixed gas is reduced to varying degrees. With the increase of temperature, the change trend of CO emission shows an exponential increase trend. In the initial stage of oxidation, the initial temperature of CO in DA, 20% CO_2_ + 80% N_2_, 40% CO_2_ + 60% N_2_, 60% CO_2_ + 40% N_2_, 80% CO_2_ + 20% N_2_ is 50, 58, 60, 63 and 73 °C, respectively. This indicates that the mixed gas can inhibit the spontaneous combustion of coal, and with the increase of CO_2_ composition, the temperature at which CO appears increases.

To quantitatively compare the inhibition effects of different gas mixtures, the inhibition rate of the whole process was calculated. The greater the inhibition rate, the stronger the inhibition effect. Calculation formula of inhibition rate:

1 $${\text{R}}=\frac{{\text{A}}-{\text{B}}}{{\text{A}}}\times 100\mathrm{\%},$$

where R is the inhibition rate of mixed gas, %; A is the volume fraction of CO produced by coal samples treated in dry air at a certain temperature; B is the volume fraction of CO produced by treating coal samples with mixed gases of different components at the same temperature.

From the above experiments, it can be seen that the greater the concentration of CO_2_ in the mixed gas, the stronger the ability to inhibit coal oxidation, and the closer it is to pure CO_2_ gas. The adsorption capacity of coal to gas is CO_2_ > CH_4_ > O_2_ > N_2_^24–27^. When the volume fraction of O_2_ is equal, the stronger the adsorption capacity of coal to other components, the weaker the adsorption capacity of O_2_ and the decrease of oxygen absorption. The adsorption capacity of coal to CO_2_ is greater than that of N_2_. Therefore, for the same O_2_ volume fraction, the greater the CO_2_ concentration in the mixed gas, the more O_2_ adsorption positions are occupied. Therefore, macroscopically, the higher the concentration of CO_2_ in the mixed gas, the higher the initial formation temperature of CO index gas in the system, and the corresponding less CO production.

**Figure 3:**
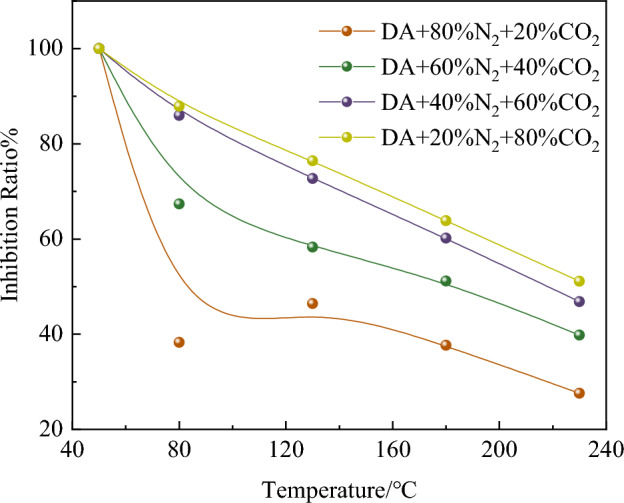
Inhibition rate of coal samples under different oxidation conditions.

Figure 3 shows that at 80 °C, the inhibition rates of coal samples are 38.88%, 55.69%, 72.73% and 81.35% respectively under 20% CO_2_ + 80% N_2_, 40% CO_2_ + 60% N_2_, 60% CO_2_ + 40% N_2_ and 80% CO_2_ + 20% N_2_. It can be seen from Fig. 3 that the inhibition rate of 80% CO_2_ + 20% N_2_ coal sample is the highest.

However, when the CO_2_ content is 60% and 80%, the inhibition ability of the mixture of the two components is very close, which shows that when the CO_2_ content in the mixture exceeds 60%, the influence on the oxidation ability of coal is further reduced, and it is advisable to add N_2_ into pure CO_2_ gas appropriately to inhibit the spontaneous combustion of coal.

### ESR spectrum change

#### ESR spectrum analysis

**Figure 4:**
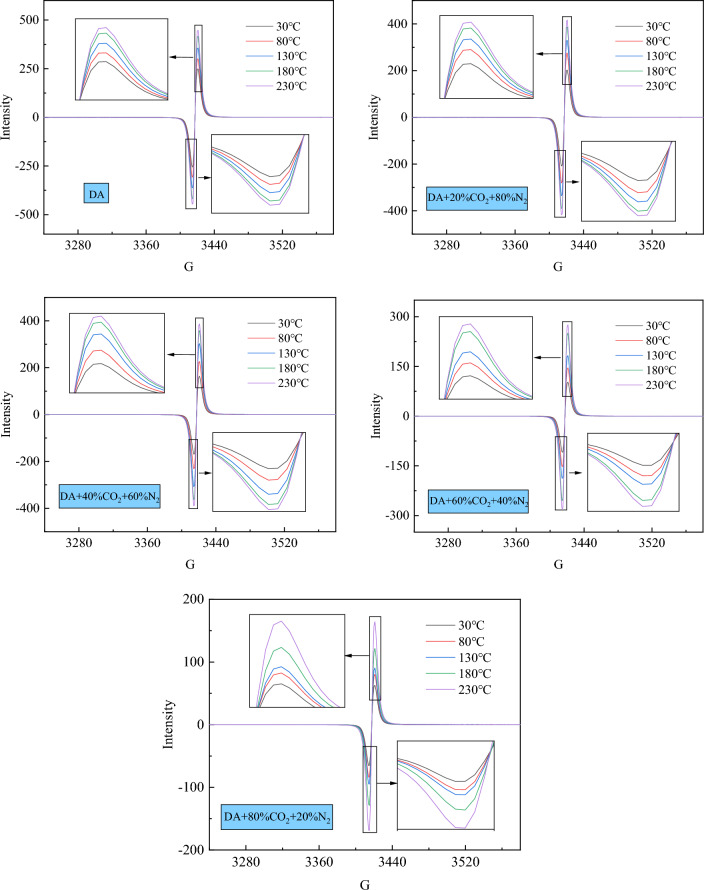
ESR spectra under different oxidation conditions.

In order to further study whether the free radical concentration in the process of coal-oxygen reaction is affected by the mixed gas, the free radical changes of coal samples under the condition of different components of mixed gas are obtained by ESR experiment, as shown in Fig. 4.

#### Free radical concentration

**Figure 5:**
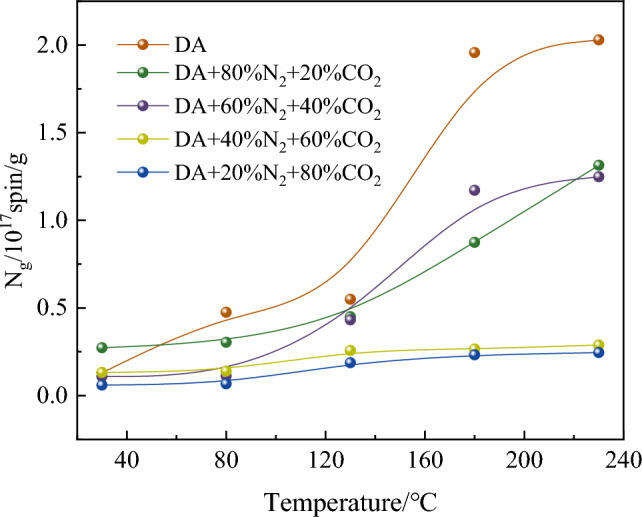
Changes of N_g_ under different oxidation conditions.

According to the experimental results, the relationship curve between free radical concentration and oxidation temperature under different oxidation conditions is obtained, as shown in Fig. 5.

It can be seen from Fig. 5 that under different oxidation conditions, the change law of free radical concentration with temperature is different. This indicates that the rate of free radical consumption is lower than the rate of free radical formation in the temperature range of 30–230 °C, showing an overall upward trend. When the CO_2_ content is less than 60%, the temperature has a great influence on the free radical reaction and the increase of free radical concentration. The experimental results show that in the whole heating stage, the total increase of free radical concentration decreases with the increase of CO_2_ ratio. Under the oxidation conditions of DA, 20% CO_2_ + 80% N_2_, 40% CO_2_ + 60% N_2_, 60% CO_2_ + 40% N_2_ and 80% CO_2_ + 20% N_2_, the free radical concentration increased by 0.19, 0.10, 0.11, 0.02 and 0.02, respectively. This is because with the further increase of temperature, the coal-oxygen recombination reaction is more intense, which makes coal enter the stage of rapid oxidation, and further generates new free radicals on the surface of coal, which promotes the chain reaction of free radicals and generates more free radicals. When the temperature reaches 230 °C, the free radical concentration of 20% CO_2_ + 80% N_2_, 40% CO_2_ + 60% N_2_, 60% CO_2_ + 40% N_2_, 80% CO_2_ + 20% N_2_ decreases by 35.25%, 38.50%, 85.80% and 87.93%, respectively, compared with that of DA, and there are obvious differences between 20% CO_2_ + 80% N_2_ and 60% CO_2_ + 40% N_2_. The reduction rate is obviously larger, because with the increase of CO_2_ concentration, it is more difficult for coal to enter the rapid oxidation stage and inhibit the free radical chain reaction of coal, so less free radicals are generated.

### g-value

**Figure 6:**
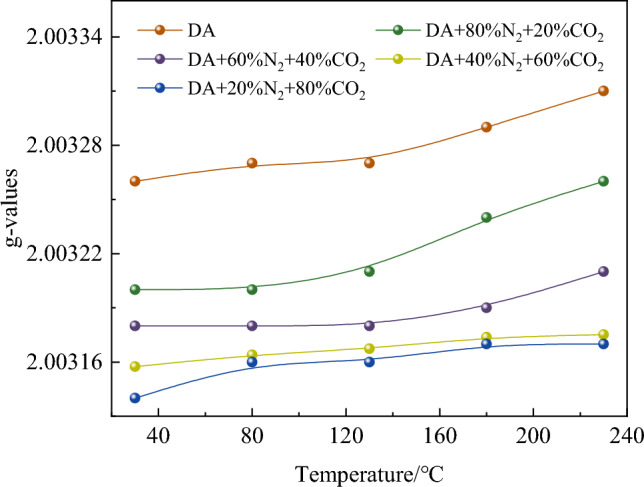
Changes of g-values under different oxidation conditions.

The g-value represents the type of free radicals, which can reflect the strength of spin–orbit coupling, that is, unpaired electron exchange during coal oxidation^28–30^. The change of g-value during coal oxidation can also reflect the change of free radicals during coal spontaneous combustion. The change rule of g-value is shown in Fig. 6.

It can be seen from Fig. 6 that under different oxidation conditions, the g-value increases slowly with the increase of temperature in the whole heating stage, indicating that the g-value is less affected by temperature. With the increase of temperature, the free radicals in coal have complex chemical changes, which lead to the change of free radicals and their types. In addition, at 230 °C, the value of g-value at DA is 0.00014 larger than that at 80% CO_2_ + 20% N_2_, which indicates that the value of g is changed by the change of gas composition. The mixed gas with different components will affect the contact chance between coal and O_2_, and then affect the generation and transfer of free radicals during coal oxidation, resulting in the change of orbit-spin coupling.

### Changes of active functional groups

**Figure 7:**
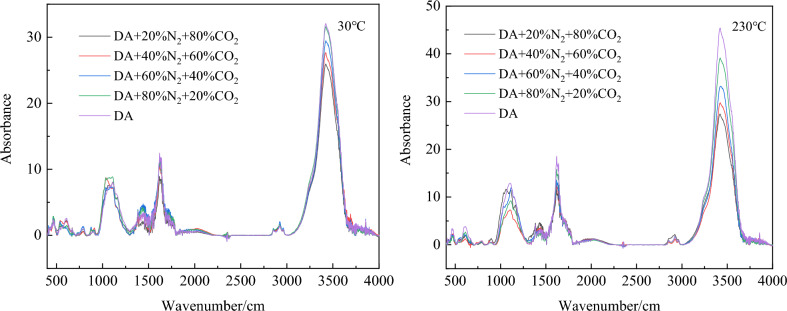
Comparison of FTIR of coal samples with different components of mixed gas.

Active functional groups in coal play the role of free radical initiation and transfer in the process of spontaneous combustion chain reaction of coal^31–33^. The changes of functional groups in coal are analyzed from the microscopic point of view, which provides theoretical support for analyzing the inhibition of inert gas mixtures with different components on coal oxidation at low temperature. In this section, FTIR spectra of raw coal and coal samples treated by mixed gas of different components at different temperatures are obtained by Fourier infrared spectra experiment. FTIR spectra at 30 °C, 80 °C, 130 °C, 180 °C and 230 °C were used to analyze the change of active functional groups in coal with temperature and the influence of different components of mixed gas on the microscopic functional groups of coal samples. Figure 7 is a comparison diagram of FTIR spectra of coal samples with different components of mixed gas at 30 °C and 230 °C.

**Figure 8:**
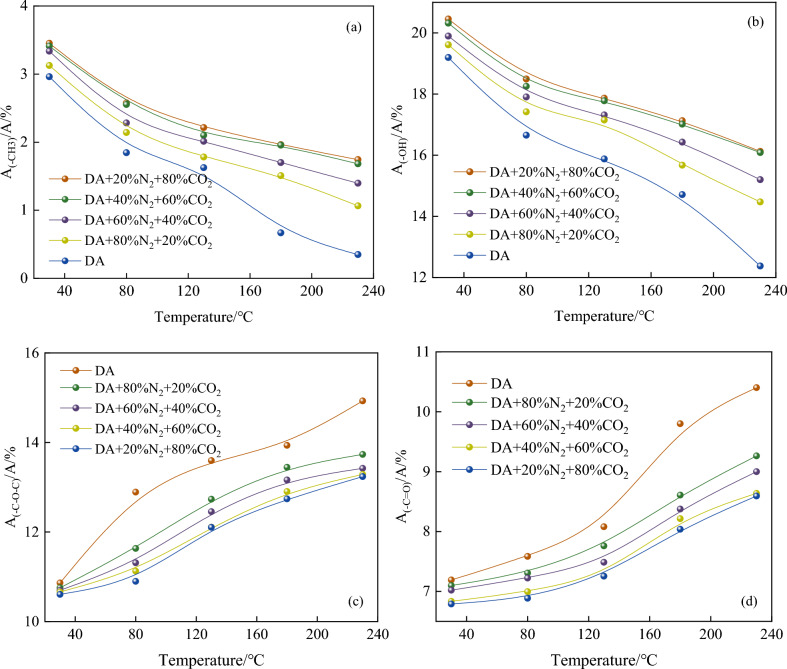
Changes of functional groups under different oxidation conditions.

It can be seen intuitively from Fig. 7 that as the temperature rises, The intensity of infrared absorption peaks of coal samples at different wavelengths is different, among which there are four different vibration intervals^34–36^: –OH absorption stretching vibration interval (3600–3000 cm^-1^), aliphatic –CH_3_ absorption stretching vibration interval (3000–2800 cm^−1^), aromatic C=O compound stretching vibration interval (1800–1500 cm^−1^) and alcohols, phenols, and ethers –C–O–C stretching vibration interval (1300–1000 cm^−1^). To more accurately analyze the changes of each functional group at different temperatures, Peakfit software is used to fit the peaks of different vibration intervals of infrared spectrum, and the peak areas of each stretching vibration interval corresponding to active functional groups at different temperatures are calculated. The results are shown in Fig. 8.

#### –CH_3_ change

Figure 8a is the curve of functional group content of coal samples with different components of mixed gas at different temperatures. As can be seen from Fig. 8a, with the increase of temperature, –CH_3_ show a decreasing trend in different atmospheres. This shows that the generation rate of –CH_3_ is less than the consumption rate. The main reason is that –CH_3_ come into contact with O_2_, undergo chemisorption reaction, are oxidized and exist in the form of peroxides. At the same temperature, with the increase of CO_2_ composition, the peak area ratio of –CH_3_ increased significantly. It can be seen that CO_2_ and N_2_ inhibit the consumption of –CH_3_ in the oxidation process. Because N_2_ and CO_2_ preempt the adsorption position of O_2_, the content of O_2_ adsorbed on coal decreases continuously, and the ability of O_2_ to attack aliphatic hydrocarbons decreases, resulting in the increase of –CH_3_ contents. With the increase of temperature, long-chain aliphatic hydrocarbons in coal decompose into short-chain aliphatic hydrocarbons, so the peak areas of –CH_3_ decrease slowly between 80 and 130 °C. When the temperature rises further, CO_2_ and N_2_ will be desorbed, and low-temperature oxidation will occur after coal and O_2_ come into contact again, which will lead to the acceleration of coal heating. When the temperature of coal rises, chemical adsorption chain reaction is formed in coal. Injection of N_2_ and CO_2_ at high temperature will dilute O_2_ concentration in coal, thus reducing the chemisorption reaction between coal and O_2_, resulting in a decrease in the formation of –CH_3_, thus inhibiting the free radical chain reaction of coal.

#### –OH change

As shown in Fig. 8b, under different conditions, with the increase of temperature, the area ratio of –OH shows a decreasing trend. Similar to –CH_2_ and –CH_3_, –OH is also the main reactive group in the process of coal oxidation. Under the conditions of DA, 20% CO_2_ + 80% N_2_, 40% CO_2_ + 60% N_2_, 60% CO_2_ + 40% N_2_, 80% CO_2_ + 20% N_2_, the reduction rates of –OH content were 21.16%, 20.82%, 23.60%, 26.20% and 35.52%, respectively. During oxidation, –OH reacts with H atoms to form water, and peroxides formed by low-temperature oxidation are decomposed and formed continuously. With the increase of oxidation temperature, water evaporates continuously, which intensifies the binding reaction between –OH and hydrogen atoms. Therefore, the consumption rate of –OH is greater than its production rate, which leads to the rapid decrease of –OH content. When the temperature reaches 230 °C, the content of –OH at 20% CO_2_ + 80% N_2_ is 0.037 higher than that at 80% CO_2_ + 20% N_2_. Under 60% CO_2_ + 40% N_2_ and 80% CO_2_ + 20% N_2_ oxidation conditions, the change trend of -OH content is similar. With the increase of CO_2_ component, the area ratio of –OH increases, which indicates that the mixed gas can inhibit the consumption of –OH in the oxidation process. Because the mixed gas preempts the adsorption position of O_2_ on coal and dilutes the concentration of O_2_, the peroxide formed by low-temperature oxidation decreases, and then the content of –OH decreases. Therefore, the oxidation reaction of coal is inhibited.

#### –C–O–C change

It can be seen from Fig. 8c that the area ratio of –C–O–C increases with the increase of temperature in different atmospheres. With the increase of oxidation temperature, the oxidation rate of coal accelerates, and some –C–O–C functional groups are formed by the reaction between C atoms and O atoms, which leads to the rapid increase of –C–O–C content. At any temperature, the area ratio of –C–O–C is always much higher than that of –C–O–C in the environment of oxidation in dry air, which shows that the mixed gas inhibits the formation of –C–O–C functional groups. When the concentration of CO_2_ is higher than 40%, it has little effect on the formation process of –C–O–C functional groups. The content of –OH is reduced by injecting mixed gas, which leads to the decrease of –C–O–C production and inhibits the coal-oxygen composite reaction.

#### C=O change

As shown in Fig. 8d, under five environments, the C=O area ratio of each coal sample increased during the heating process. In the initial stage of oxidation, part of C=O is oxidized to form –COOH, which leads to slow increase of C=O area ratio. With the low temperature oxidation reaction, –CH_2_, –CH_3_ and –OH in coal undergo oxidation reaction, and more C=O groups are formed. At 230°C, the content of C=O at 20% CO_2_ + 80% N_2_ is 0.67 higher than that at 80% CO_2_ + 20% N_2_, which indicates that CO_2_ composition affects the formation of C=O. The higher the CO_2_ content, the lower the C=O production. This is because the mixed gas affects the production of –CH_2_, –CH_3_ and –OH, which leads to the lower production of C=O, thus reducing the free oxygen-containing active radicals in coal and inhibiting the oxidation activity of coal.

### Mechanism of inert gas mixture affecting free radical reaction

To further understand the adsorption behavior, three adsorption sites on the cluster model are considered: the top (T), the top of the C atom; Key (B), the midpoint of the C–C bond; Center (C) is the center of the hexagonal hexagonal aromatic cluster. For each position, two adsorption directions of CO_2_, including vertical (V) and parallel (P), were used to obtain eight adsorption models of CO_2_ on the Gr surface^32^. By comparison, the most stable position of CO_2_ adsorption on the model was found.

**Figure 9:**
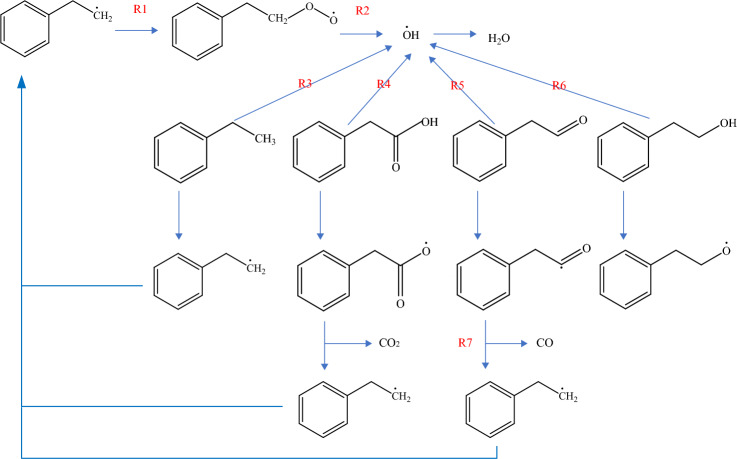
Chemical adsorption reaction process of coal.

The mixed gas not only hinders the adsorption of O_2_ by coal, but also affects the activation energy and exothermic conditions of the chemical adsorption of O_2_ by coal, thus affecting the reaction process. The chemical adsorption reaction process of coal is shown in Fig. 9^37,38^.

**Figure 10:**
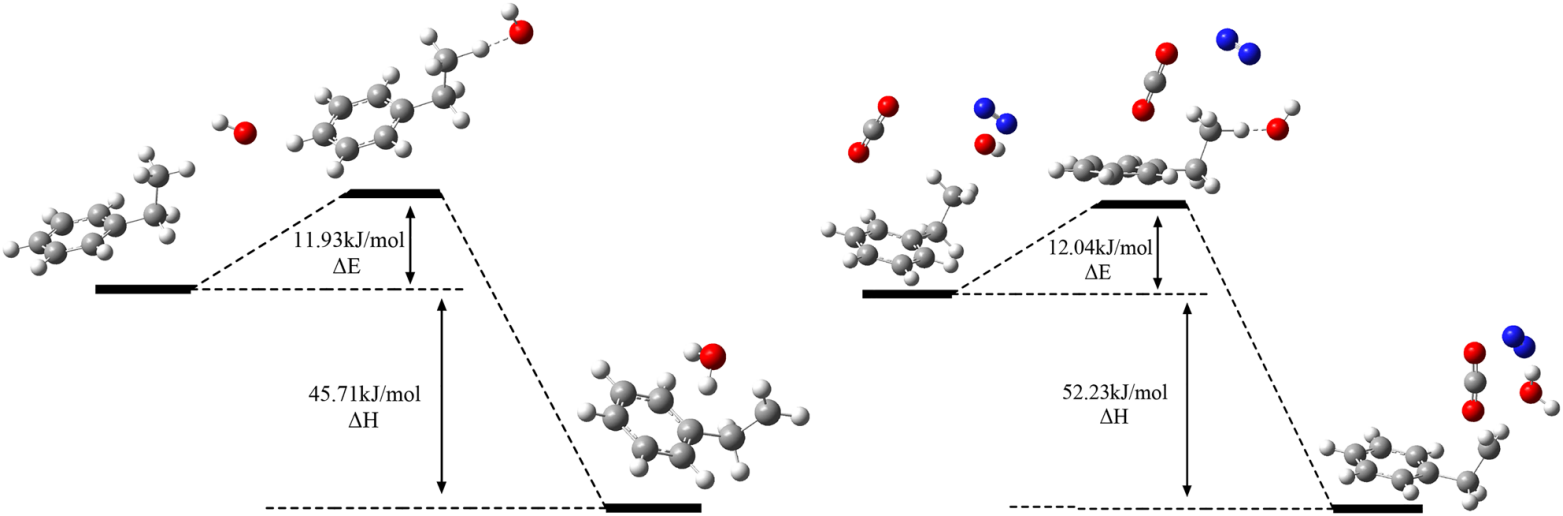
Reaction mechanism of R3 with and without mixed gas.

**Figure 11:**
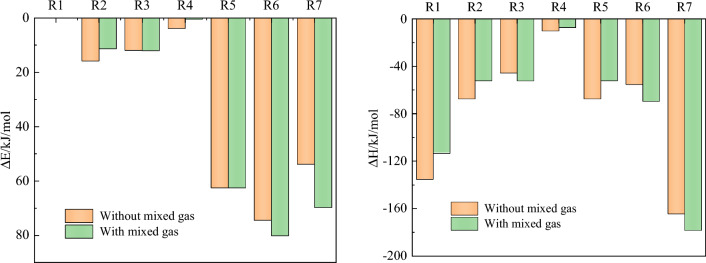
Activation energy and heat change with or without mixed gas.

The above reactions were simulated with and without mixed gas, respectively. The reaction process of R3 with and without mixed gas is shown in Fig. 10. The changes of activation energy and heat are shown in Fig. 11. R1 is the chemisorption of O_2_ and does not need activation energy. The heat release decreases from 135.26 to 113.48 kJ/mol. R2 is a reaction that produces hydroxyl radicals. The activation energy decreases from 15.85 to 11.37 kJ/mol; Its heat release decreased from 67.38 to 52.19 kJ/mol. R3–6 is that the active group in chemisorption state adsorbs the surrounding hydrogen atoms. The mixed gas has little effect on the activation energy of R3, and its exotherm increases from 45.71 to 52.23 kJ/mol. For R4, the activation energy decreased from 3.87 to 0.52 kJ/mol; The heat release decreased from 10.15 to 7.39 kJ/mol. The activation energy of R5 has no effect on the mixture gas. When the mixture gas is present, the exothermic heat of R5 decreases from 67.38 to 52.19 kJ/mol. For R6, the activation energy increases from 74.47 to 80.14 kJ/mol with mixed gas; The heat release increased from 55.36 to 69.53 kJ/mol. For R7, the activation energy increased from 53.86 to 69.74 kJ/mol with mixed gas; The heat release increased from 164.52 to 178.31 kJ/mol. To sum up, the mixed gas has no significant effect on R1, R3, and R5, but it will reduce the exothermic heat of R1 and R5, and then affect the subsequent reaction process. The mixed gas can promote R2 and R4 and hinder R6 and R7.

It can be seen that the mixed gas hinders the chemical adsorption reaction between O_2_ and coal surface molecules, which leads to the decrease of peroxides, so the content of hydroxyl radicals is reduced, which inhibits the chain reaction of coal radicals and plays a chemical inhibition role.

## Conclusion

Through thermodynamic experiments, testing methods and density functional theory, the low-temperature oxidation characteristics of coal under different components of mixed gas are analyzed, and the results show that the mixed gas affects the free radical reaction and then inhibits the natural oxidation of coal. The specific conclusions are as follows:

1. The results of temperature programmed experiment show that the higher the CO_2_ concentration in the mixed gas, the stronger the inhibition ability of coal oxidation. But when the CO_2_ content is 60% and 80%, the inhibition ability of the mixed gas of the two components is very close. It is advisable to add N_2_ into pure CO_2_ gas properly to inhibit coal spontaneous combustion.
2. The influence of mixed gas on the concentration and species of free radicals was obtained by ESR experiment. When the temperature reaches 230 °C, the free radical concentration of 20% CO_2_ + 80% N_2_, 40% CO_2_ + 60% N_2_, 60% CO_2_ + 40% N_2_, 80% CO_2_ + 20% N_2_ decreases by 35.25%, 38.50%, 85.80% and 87.93%, respectively, compared with that of DA.
3. Through FIIR experiment, the change of functional group content of mixed gas with different components at different temperatures was analyzed, and it was found that the larger the CO_2_ component in the mixed gas, the lower the amount of –OH generated, and then the increase of oxygen-containing functional group content. However, when the CO_2_ content is 60% and 80%, the influence of the mixed gas of the two components on the change of functional group content is close.
4. The chemical adsorption reaction between O_2_ and coal surface molecules is inhibited by quantum chemistry calculation, and the activation energy of free radicals is increased and the heat release of the reaction is reduced. Inert gas mixture inhibits the spontaneous combustion of coal by affecting the free radical reaction process.

## Data Availability

The datasets used and/or analysed during the current study available from the corresponding author on reasonable request.
